# Versatile clinical movement analysis using statistical parametric mapping in MovementRx

**DOI:** 10.1038/s41598-023-29635-4

**Published:** 2023-02-10

**Authors:** Amr Alhossary, Todd Pataky, Wei Tech Ang, Karen Sui Geok Chua, Wai Hang Kwong, Cyril John Donnelly

**Affiliations:** 1 Rehabilitation Research Institute of Singapore (RRIS), Nanyang Technological University, Singapore, Singapore; 2 Department of Human Health Sciences, Kyoto University Graduate School of Medicine, Kyoto, Japan; 3 Tan Tock Seng Hospital Rehabilitation Centre, Singapore, Singapore; 4 Department of Rehabilitation Sciences, The Hong Kong Polytechnic University, Hong Kong, People’s Republic of China

**Keywords:** Software, Rehabilitation, Osteoarthritis, Preventive medicine, Orthopaedics, Musculoskeletal system, Comorbidities

## Abstract

Clinical gait analysis is an important biomechanics field that is often influenced by subjectivity in time-varying analysis leading to type I and II errors. Statistical Parametric Mapping can operate on all time-varying joint dynamics simultaneously, thereby overcoming subjectivity errors. We present MovementRx, the first gait analysis modelling application that correctly models the deviations of joints kinematics and kinetics both in 3 and 1 degrees of freedom; presented with easy-to-understand color maps for clinicians with limited statistical training. MovementRx is a python-based versatile GUI-enabled movement analysis decision support system, that provides a holistic view of all lower limb joints fundamental to the kinematic/kinetic chain related to functional gait. The user can cascade the view from single 3D multivariate result down to specific single joint individual 1D scalar movement component in a simple, coherent, objective, and visually intuitive manner. We highlight MovementRx benefit by presenting a case-study of a right knee osteoarthritis (OA) patient with otherwise undetected postintervention contralateral OA predisposition. MovementRx detected elevated frontal plane moments of the patient’s unaffected knee. The patient also revealed a surprising adverse compensation to the contralateral limb.

## Introduction

### Clinical gait analysis

Gait analysis is generally described as a subjective analysis of waveform data augmented by high and low fidelity data through 2D and 3D instrumentation^1^. There are two types of gait analysis namely quantitative analysis and qualitative or observational analysis. quantitative analysis generally uses different motion capture instrumentations. Observational gait analysis is indeed more affordable and more accessible, yet it is neither accurate nor repeatable to be suitable for the assessment of a clinical gait population^2,3^.

Quantitative Gait Analysis is sometimes referred to as Clinical Gait Analysis (CGA) or Numerical Gait Analysis. It is a process whereby gait characteristics are measured, abnormalities are determined, causes are postulated and treatments are proposed^4^. CGA helps in the diagnosis, assessment, and treatment of several common gait diseases like cerebral palsy, Parkinson’s disease, multiple sclerosis, cardiopathies, post-stroke hemiplegic gait, neuropathic, and myopathic diseases, as well as rehabilitation and bioengineering^2,5,6^. CGA usually consists of 5 elements: videotape examination, measurement of spatiotemporal gait parameters, kinematic analysis, kinetic measurement, and electromyography (EMG). Measurements of gait parameters includes cycle time, stride length and speed. Kinematics is the measurement of the movement, and kinetics measures the force beneath each foot while walking. EMG is the measurement of the electrical activity of muscles^7^.

Traditionally, whole-trajectory analysis was conducted by the human eye as the statistical models in general were unavailable. Thus, one major limitation of traditional gait analysis is its subjectivity, being prone to both type I (false positive) and type II (false negative) errors, where the evaluator detects problems that are not present or miss already present problems respectively. It is not only highly dependent on the experience of the evaluator, but also non-coherent i.e., significantly different even among evaluators with similar experience^2^. To show the strength of statistical parametric mapping (SPM) in data continuity analysis, Donnelly and colleagues compared whole-trajectory statistics to expert qualitative assessment. Although the two experts were of a comparable level of expertise, their evaluations agreed with each other in only **61%** of the available degrees of freedom and phases. However, when their independent analyses were pooled together, the pooled assessment was **83%** in agreement with the whole trajectory analysis^2^. To mitigate this non-coherence problem, CGA is usually performed as the collective effort of a team of clinical gait experts, which is both time and cost expensive^2^.

### SPM

Statistical parametric mapping (SPM) is a method that uses Random Field Theory (RFT)^8^ to perform whole-trajectory analysis using topological inference. SPM has been primarily used in Neuroimaging^9^, but can be applied to any spatiotemporally registered and smooth signal. In the context of multivariate one-dimensional trajectories, like those measured during gait (e.g. kinematics, moments, EMG etc.), SPM can be used to quantify whole-trajectory multivariate effects^10^. One-Dimensional Statistical Parametric Mapping (SPM1D)^11^ is a Python/MATLAB package which has been employed in the analysis of a variety of human movement studies in both healthy and pathological conditions (hemiplegic stroke gait, transtibial amputee gait and knee OA) using biomechanical measurements (kinematics, moments, and EMG)^12–14^. While several other software packages implementing the SPM methodology were available (rft1d, SPM12, fmristat, and nipy), SPM1D is currently the only package designed specifically for the analysis of one-dimensional data, like the multivariate time series that are measured during gait^15^.

## This work

This paper is intended to present MovementRx, a Graphical User Interface (GUI) decision support System that provides a holistic view of all joints along the kinematic/kinetic chain of the lower limbs related to functional gait; to provide an objective assessment of locomotion for clinicians. It utilizes SPM1D combined with a visualizing software to present the magnitude of statistical effect in a simple, novel, coherent, objective, and intuitive manner.

The most important feature of MovementRx is that it is a purpose-built clinical tool with color maps to improve the interpretation of the data and reduce the need for statistical knowledge of the user. Users can identify and rapidly visualize areas within the movement profile that are deviant from normative data band or pre/post-surgical intervention, then cascade from joint to joint, and move from the 3D joint level to its 1D scalar components and back with the click of a button. To show how MovementRx can help users spot and interpret results, we will present a case where MovementRx highlighted the condition of an osteoarthritis patient who underwent knee arthroplasty, which shows an improved condition in the affected limb, but an elevated risk of comorbidity in the other limb. Detailed studies of other intended use cases will be published in different papers.

## Methods

### Implementation

We implemented MovementRx as a standalone Graphical User Interface (GUI) python application, using Python 3.7. We depend on the following libraries: “NumPy” for data structures, “SPM1D” and “SciPy” for movement-specific scientific calculations, “PyQt5” to build the GUI, and “matplotlib” to plot the graphs and colormaps.

The application—as most Qt applications- implements a modified Model-View-Controller design pattern. It is organized into the following sub packages:

- Models: It contains the DataManager class and the current and any upcoming data sources.
- Controls: It contains the Controller interface as well as windows / dialogues that implement the Controller interface.
- UI: It contains the DisplayManager interface, which is expected to be implemented by any user interface class. It includes 2 sub packages as well:
  o GUI: where all GUI classes are located. Those which are generated using the Qt builder (from XML files) are stored in a subpackage called “xml”. They are inherited by classes in the “controls” packages.
  o CLI: to be used if the user wants to interact with the application from the command line interface (CLI).
- Tools: It contains utility functions to deal with data from different sources in different formats. They include resampling the input files to time normalize data of 101 samples per gait cycle, as well as scaling data (e.g. body-weight normalizing) and flipping (sign-changing) some measurements in the event the data was not modeled using ISB recommendations or if a laboratory uses their own conventions (i.e., robotics).

### Intended use

MovementRx is developed to be a decision support system (DSS) that correctly models whole-trajectory multivariate variance. It is versatile in the sense that it can be used for the movement analysis of any clinical case study. MovementRx main use is CGA of lower limb major joints, excluding Metatarsophalangeal (MTP) and distal joints, with the ability to extend to all joints with minor amendments to the source code. Three currently active uses of MovementRx for gait analysis in our laboratory include unilateral conditions such as hemiplegic stroke gait, transtibial amputee gait and knee OA. We will provide details thereof in subsequent publications.

MovementRx is intended to be used as a GUI application. However, in its architecture, we included the placeholder for command line interface to be used should the need arise, e.g., for batch processing of multiple cases. When imported as a python package, its tools and functions can be imported as a library and be used alongside third-party applications like OpenSim^16^ or AnyBody^17^.

### Data

#### Data source

MovementRx can accept data from any high-fidelity stereoscopic system, e.g. *Vicon*, *qualysis*, as well as middle fidelity inertial sensor systems, e.g. *Xcense*. Data sampling rate however must abide by Nyquist Theorem.

### Data format

Currently, MovementRx accepts data in the form of comma separated files (.csv files), being simple to produce, interpret, and export. Later, we will implement HP5 format.

For the CSV input files, each file holds the measurements of one dimension per limb per study (moments / kinematics) per subject. Each data in every file is formatted as one header line followed by multiple lines of equal length (101 samples). Each line represents a single trial (in case of a subject) or the average of the trials of a subject in case of reference / norm. Please see www.spm1d.org for more information on data formatting.

#### Normative data

The normative (reference) data comes from 20 participants’ recordings from the ability data project^18^. They were all males, aged 55–75. The summary of their biomechanical measurements is presented in Supplementary Table 1. These participants were chosen as the walking velocity, age, gender, and BMI align with the case study participant. For Ethical approval and consent details, please refer to section (Ethics approval and consent to participate).

### GUI overview

**Figure 1:**
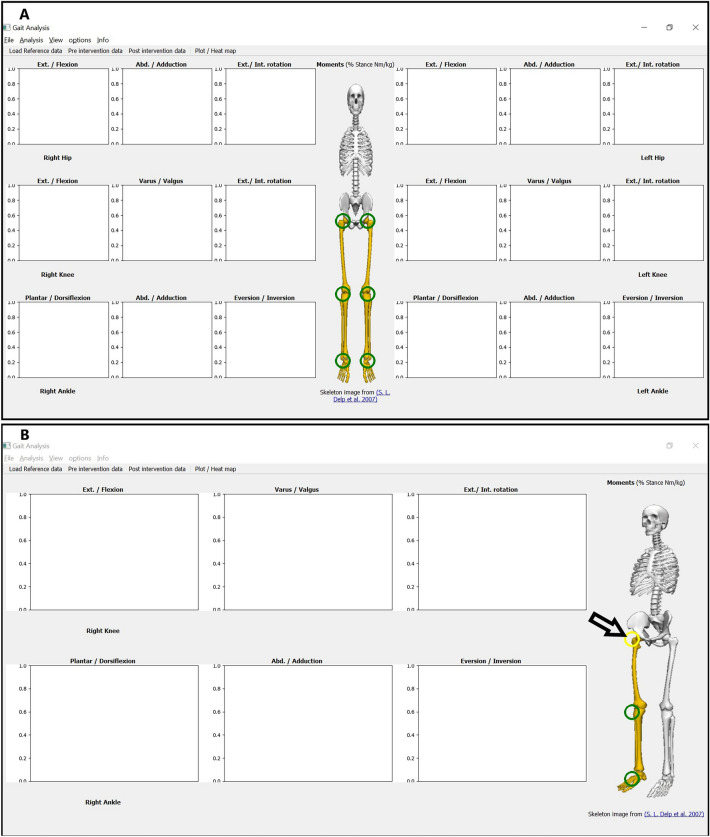
Initial MovementRx main window. (**A**) Main window at start up. (**B**) Focused view on 2 joints (right knee and ankle) only, hiding the whole left side and right hip. The skeleton image is used as an approximate visual reference, to emphasize the currently visualized limbs/joints.

When the application is in the “gait analysis” mode (default), the main application window (Fig. 1A) shows a skeleton (in the middle), that separate between two (left and right) composite panel views. Each side represents one leg view. Each leg view is divided from top to bottom into three joints: hip, knee, and ankle. The movements of each of the three joints can be analyzed as 3D vector or further divided into its three 1D components: e.g. flexion/extension, adduction/abduction, and internal/external rotation in case of the knee joint. The naming of each dimension is ordered such that the positive direction comes first (e.g. Ext./Flexion means that the extension movement in that joint is considered positive while the flexion movement is considered negative). To help the user to focus on the joints of interest (those with statistically meaningful results), the user can choose to hide / show one side of the body from the “view” menu, and to hide / show a particular joint by clicking on the joint of interest on the skeleton. When a joint is hidden, the button is hidden as well, and it appears as in yellow when the mouse hovers it. Fig. 1B shows the application after hiding the left side, then hiding the right hip by clicking the relevant joint button. An arrow in the figure indicates where to click to hide the joint.

**Figure 2:**
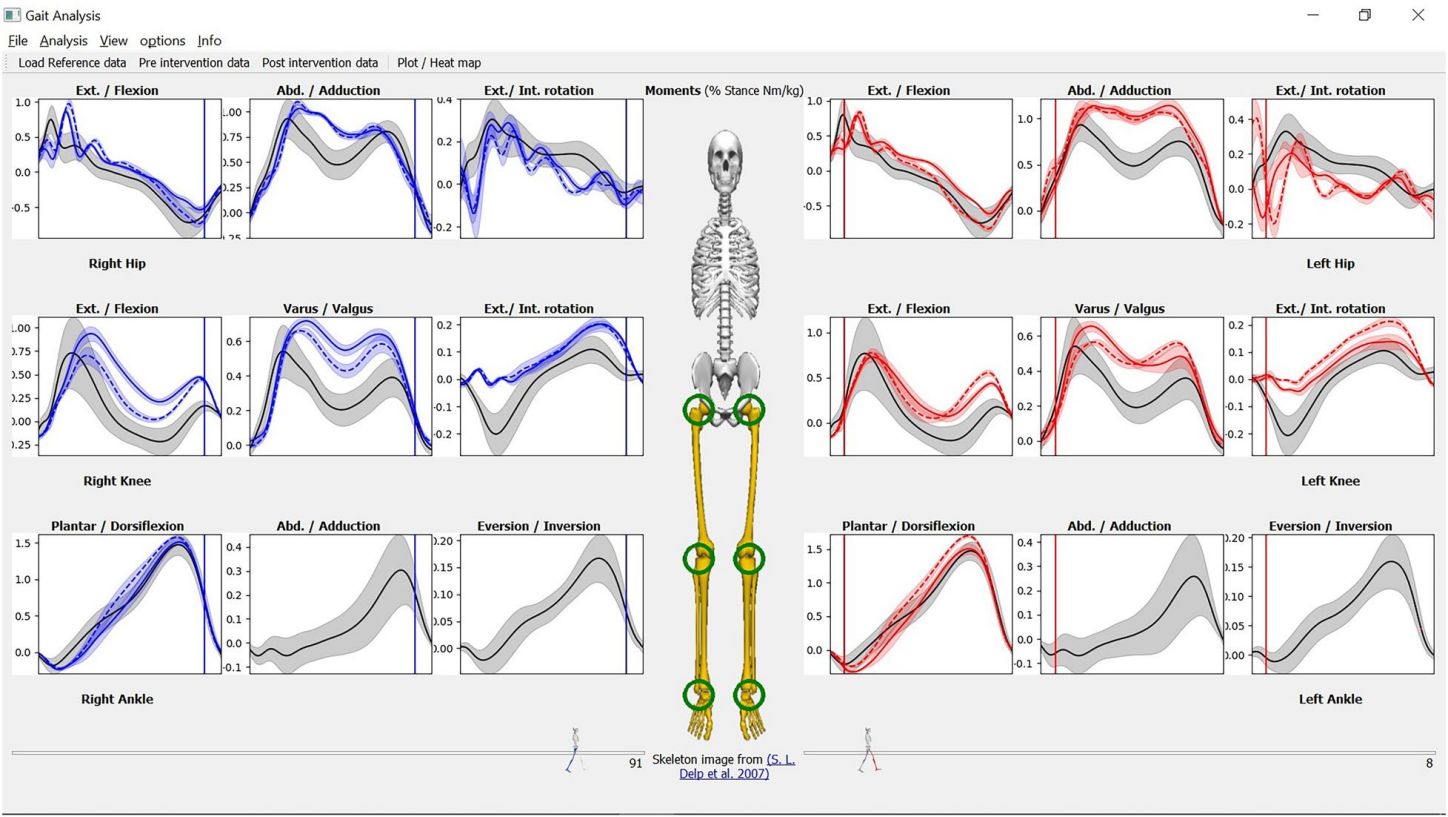
MovementRx after loading the reference, pre and post intervention data. The black line is the reference, the solid color line is the preintervention data, while the dashed line is the post intervention data. The pre and post intervention data are colored blue for the right limb and red for the left limb. The same color code applies for the animated walking men below and related indicator in the panels. This recording does not have ankle values for both Y (adduction/abduction) and Z (inversion/eversion) dimensions due to the use of a simplistic model not designed for 3D ankle mechanics.

As shown in Fig. 2, there are three sets of recording data files that can be loaded and viewed: (1) *Reference* (in black solid lines), (2) *preintervention* (in solid lines), and (3) *postintervention* (in dotted lines). The right side is colored in blue while the left side is colored in red for both pre and post intervention. Any combination of the three sets can be loaded in any order. Each set of the three can contain both kinetics and kinematics records. The initial view (in Fig. 2) shows the *moments* view. The *kinematics* view is shown in the “Kinematics view” section.

We provide at least one default reference with the application; the details thereof were provided in the “Normative data” section. However, it is the user’s responsibility to select the reference that most appropriately matches the age, race, gender, etc. of the case under assessment. Figure 2 depicts a case of Total Knee Replacement (TKR) on the right side. The details of this case will be discussed in “The presented case study” section later.

#### Animated gait

At the bottom of the window, there are two walking skeletons—one for each limb—showing the stage in a full stride (kinematics) from heel contact to heel contact of the same limb; or stance (kinetics) from heel contact to toe off. When they are animated, moving relevant vertical lines appear on the graphs. This allows the user to have a synchronized view on anomalies/deviations among the three dimensions in different stages of the gait cycle.

### Available analysis methods

The availability of analysis method is based on the availability of loaded data from $\{$reference, preintervention, postintervention$\}$. All analysis modalities are available from the analysis menu, the user can choose the method of analysis the user is interested in, from the following list of options:

- *Pre-intervention vs Reference* The 3D vector is compared using Hotelling’s 2 test, while the individual components are compared using T test 2 (Two-sample t test).
- *Post-intervention vs Reference* The 3D vector is compared using Hotelling’s 2 test, while the individual components are compared using T test 2.
- *Pre- and Post- intervention together vs Reference* (this combines the abovementioned two modes in the same view): The 3D vector is compared using Hotelling’s 2 test, while the individual components are compared using T test 2.
- *Pre vs. Post* Comparing the status before intervention versus its counterpart after intervention, irrespective to a third reference. There are two modes for such a comparison:
  o *Paired* The 3D vector is compared using Hotelling paired test, while the individual components are compared using paired T test.
  o *Two Sample* The 3D vector is compared using Hotelling’s 2 test, while the individual components are compared using T test 2.

**Figure 3:**
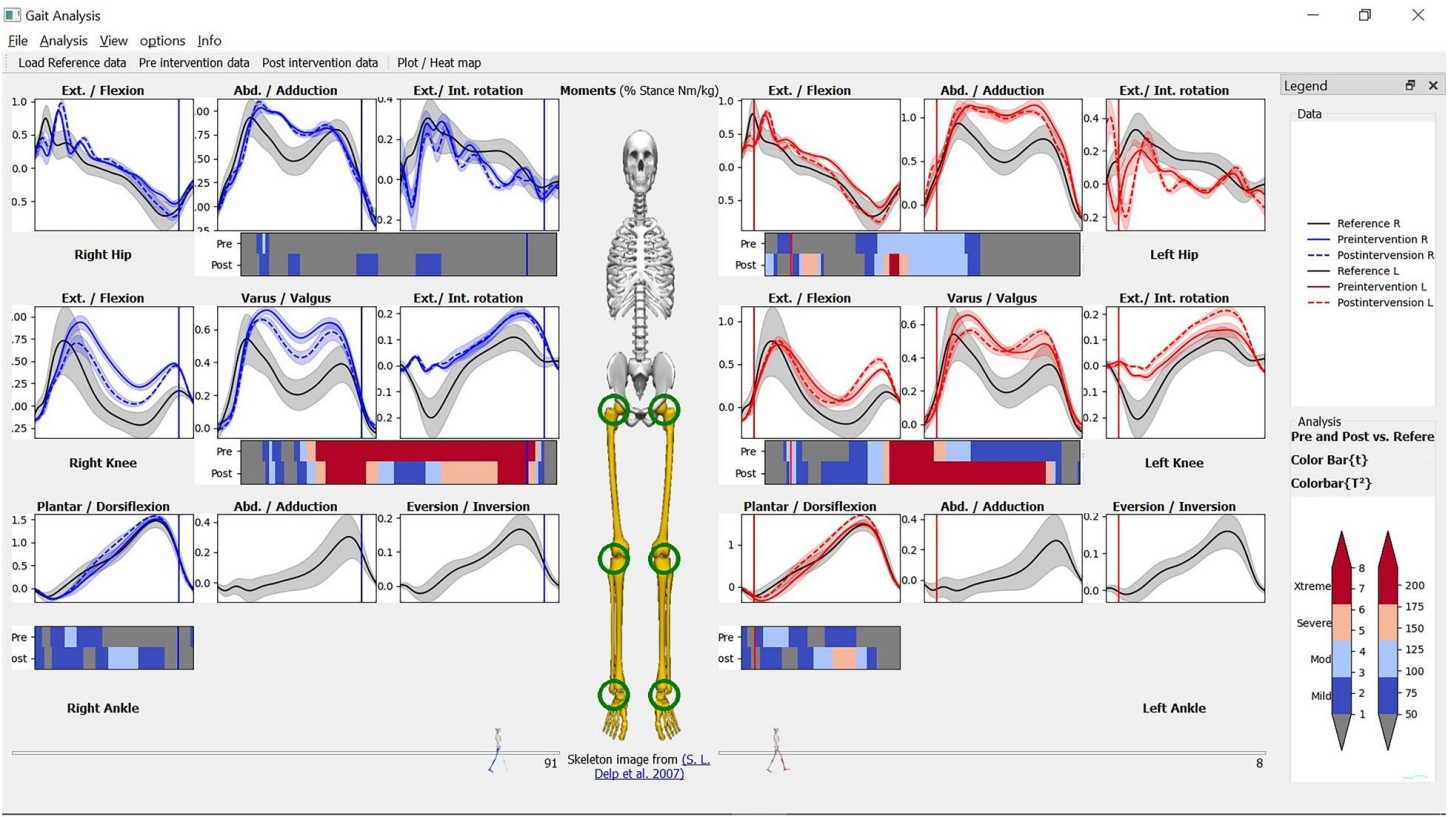
MovementRx after applying pre and post intervention vs reference Hotelling’s 2 test.

After selecting the analysis method, a combined colormap representing the combined severity of the abnormalities vector in all three dimensions appears below every joint in the view panel. Figure 3 shows a screenshot of MovementRx after applying pre and post intervention vs reference.

The legend present in the right side of Fig. 3 can be shown, hidden, or undocked to float as a separate window. The severity of the deviation of the case from the reference normal is color-coded from blue (subtle/mild) to dark red (extreme/very severe), scaling from 1 to 4 folds in case of individual components (left) and from 1 to 8 folds in case of the combined 3D bar (right). The legend can be docked to either side of the application, float undocked as a separate window, or be hidden altogether. Supplementary Fig. 1 shows the legend in the docked state.

The user can then cascade down to view the analysis of individual components of each joint, by clicking the respective bar. Clicking the bar will show / hide three bars, for the three respective dimensions.

**Figure 4:**
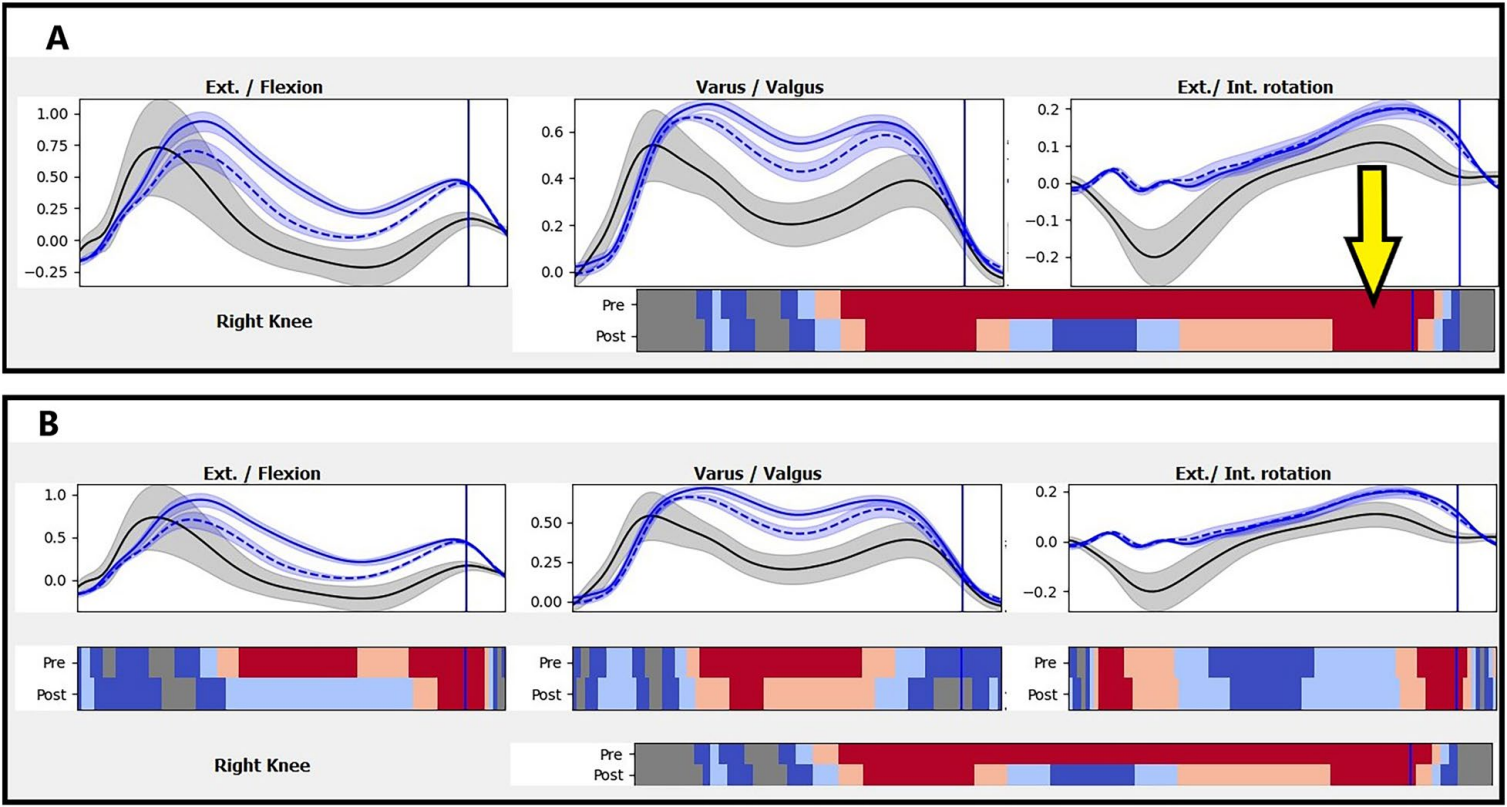
Close view of the right knee. (**A**) with hidden individual components. 1st row shows the recordings (reference, pre, and post intervention) in 3 dimensions. 2nd row shows the result of comparisons of both the preintervention vector (above) and postintervention vector (below) to the reference data vector, using Hotelling’s 2 test. The yellow arrow points to the 3D colormap bar. Clicking it will toggle the 3 individual components panels shown / hidden. (**B**) Same view of (**A**) after showing individual components. Upper and lower rows: as in (**A**), Middle row: Appearance of the result of comparison of individual components for both preintervention (above) and postintervention (below) to the reference data, using T test 2.

Figure 4 show a zoomed in view of the right knee panel from Fig. 3 (pre and post intervention vs. reference) with hidden and shown individual components, respectively.

### Other options

#### Alpha

The Type I error rate (alpha) is conventionally 5% but MovementRx allows the user to choose the alpha from several pre-set alpha values, or to write their own preferred value. Supplementary Fig. 2 shows the alpha option in options menu.

#### Colour scale

**Figure 5:**
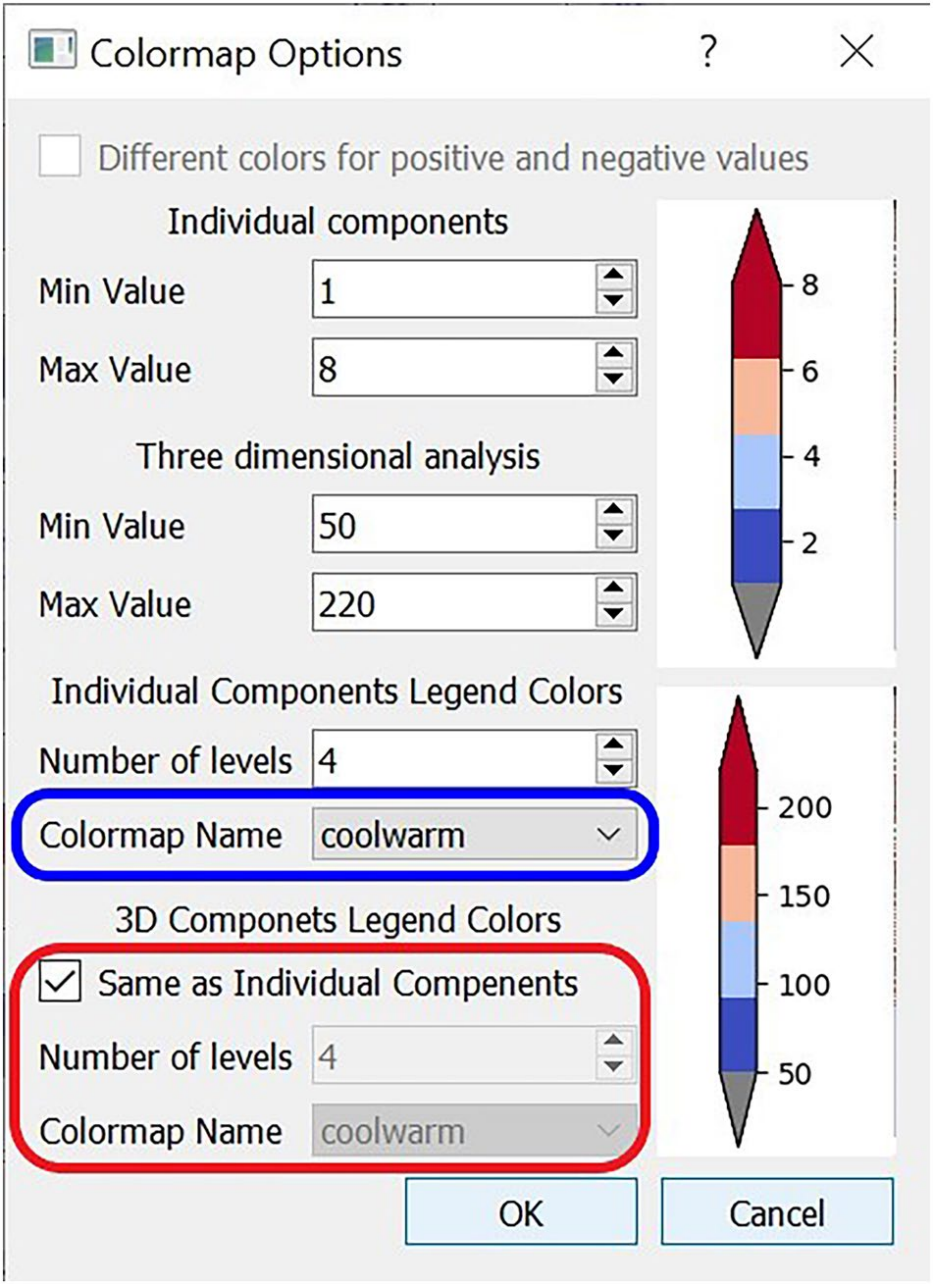
Color Scale dialogue.

Sometimes, the clinician wants to highlight some parts of the severity scale, or to use a different colour map for logistic reasons. Colour scale menu item allows changing the colour scheme and parameters of the colormap in one place. Figure 5 shows the colour scale dialogue with 4 sections:

1. Individual components to change their minimum and maximum values.
2. Three-dimensional analysis to change their minimum and maximum values.
3. Individual components legend colours to adjust the number of levels on the colour bar and to select one colormap from a list of 3 common MATLAB-like colormaps (cool to warm, jet and viridis) available by default, as shown on the figure highlighted in blue.
4. 3D components legend colours: By default, it is set as the same as Individual components, but the user can change the colormap colours, as shown on the picture highlighted in red.

### Other views

#### Plot view of individual components

**Figure 6:**
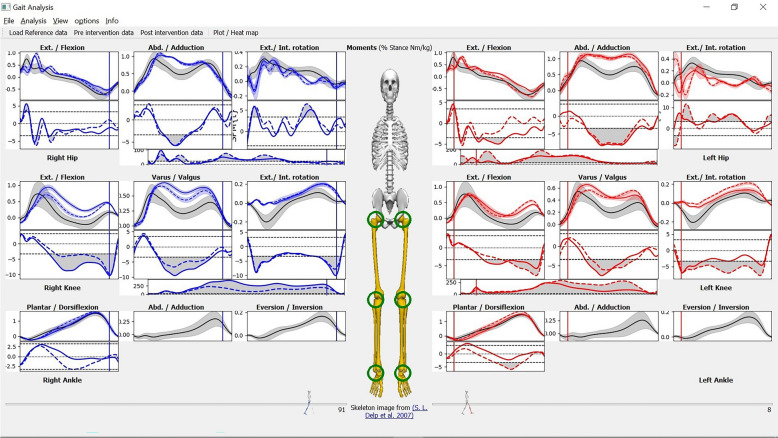
Plot of individual components, showing the same case of Fig. 3 after switching to the plot view.

For the sake of scientific interest, the user can view the individual components of movement as an SPM1D plot (spm1d.org)^11,13^. Figure 6 shows the same data and analysis results of those from Fig. 3, after switching to plot view. This option is available from the view menu and from the toolbar below it.

#### Kinematics view

**Figure 7:**
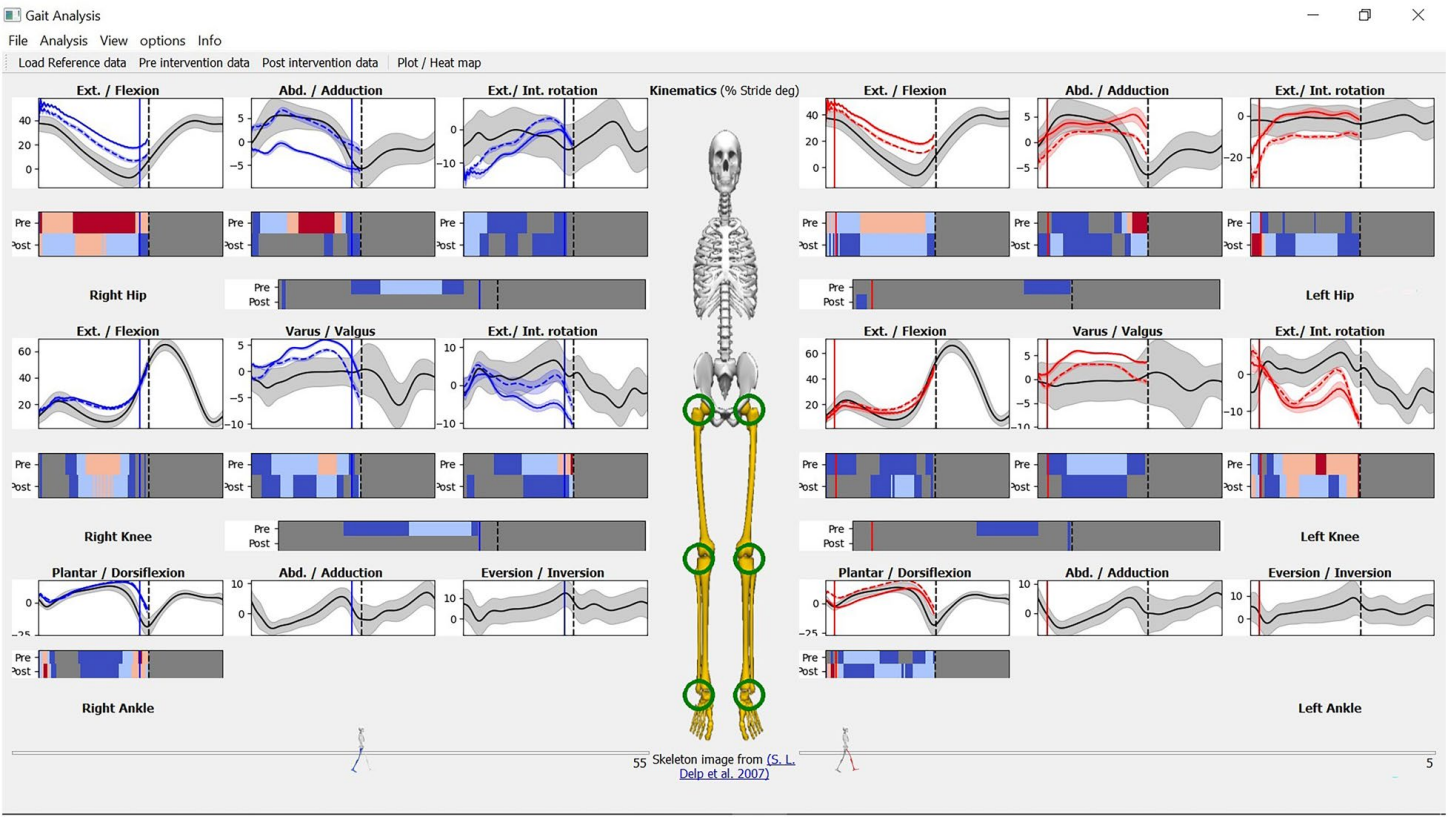
Kinematics View.

From the View menu, the user can choose to switch between the moments and kinematics views. Figure 7 shows the kinematics view. It shows identical features to those available in the Moments view.

### Ethics approval and consent to participate

Ethics approvals were granted by the Human Research Ethics Committees of the University of Western Australia (RA/4/1/6088) and St John of God Hospital (#802 and #880). Ethics approval for data collection of reference norm data was granted as part of “Mobility Data” project. All methods were carried out in accordance with relevant guidelines and regulations. Informed consent was obtained from all subjects.

## Results and discussion

### The presented case study

Here, we present one case of interest. The presented case is a 55-year-old male (height: 1.73 m, weight: 77 kg) who suffered from Kellgren-Lawrence Classification System grade 3 knee osteoarthritis in the right knee. He had undergone total knee replacement (TKR). The pre-surgery gait analysis was conducted 1 month before the surgery. The Pain subscale score of the Knee Injury and Osteoarthritis Outcome Scores was 44.4. The post-surgery data collection was conducted 1 year after the surgery. The pain score was 77.8. No additional assessment was conducted in between the two data collections. We recorded 7 ‘10-m walk’ trials within the testing session. A “trial” is one walking/recording bout involving uninterrupted straight-line walking at comfortable speed. The participant was instructed to simply: “walk at a comfortable speed”. A 16-camera VICON motion-capture system (VICON Motion System Ltd, Oxford, UK) was used to record three-dimensional markers trajectories. The marker set used in the current study has been described in a previous study^16^. A pipeline in Visual 3D Professional (v2021.04.01; C-Motion, Inc., Germantown, MD, USA) was then used to process all dynamic trial data. Briefly, the centres of the ankle joints were defined as the midpoint of the medial and lateral malleolus, while the centres of the knee joints were defined as the midpoint of the medial and lateral epicondyles. The hip joint centres were estimated in the CODA pelvis segment. Two force platforms (Type 9260AA6, Kistler, Switzerland) recording at 2000 Hz were used to record the ground reaction force. The two force plates were embedded in the ground. They were placed end-to-end and recorded consecutive strides. They were visible to the participant -as we were unconcerned with visibility- because visibility is unexpected to affect the left and right limbs differentially. The net internal moment at the hips, knees and ankles joints were resolved in the local coordinate system of the proximal segment using the inverse dynamics analyses of kinematics. Kinematic trajectories and ground reaction forces were both filtered with a zero-lag fourth-order low pass Butterworth filter at 6 Hz, which was selected using residual analysis and visual inspection of the kinematic data.

The recording was then compared to 20 healthy individuals as a control group. The post intervention data was recorded 1 year after the TKR he had. The control (reference) model matches the patient’s age group and gender. A detailed description of the procedure of data acquisition in a “10 m walk” task is described in a previous paper^18^.

We loaded both pre and post intervention and the reference model in MovementRx as shown in Fig. 2. Then we applied the “pre and post intervention vs reference” analysis. Figure 3 is a screenshot of the application after showing the analysis results. Fig. 4A,B show close views of the right knee (the affected knee) with the individual components hidden/shown, respectively.

MovementRx is meant to accept data from different sources. Some data recordings or parts thereof may be missing in some cases. Unfortunately, the ankle data in the Y (adduction/abduction) and Z (inversion\eversion) dimensions for this patient are not available. However, the application just bypassed the missing data and worked without crashes. Likewise, the kinematics of the last 40% of the patient’s gait cycle is missing. However, the application just ignored it and worked without crashing, as shown in Fig. 7.

#### Findings and interpretation

Figure 3 shows MovementRx after loading the data before and after the intervention and applying “pre and post intervention vs reference” Hotelling’s 2 test view. It shows a clear reduction of the moments on the right knee (post-TKR), especially in the X (flexion/extension) and Y (adduction/abduction) components as detailed in Fig. 4B.

**Figure 8:**
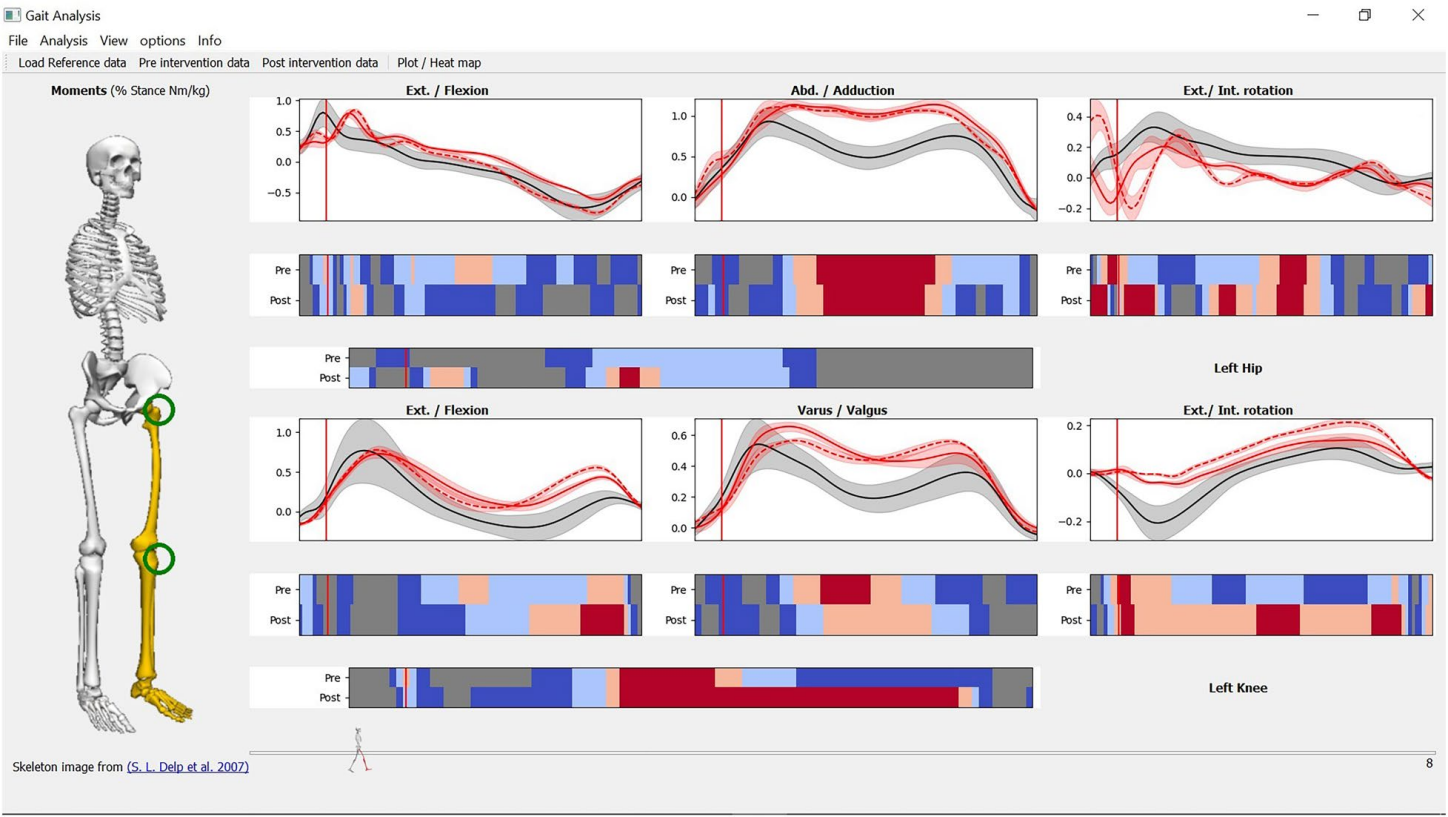
Left hip (upper) and knee (lower) only after hiding the ankle and applying pre and postintervention vs reference analysis to the hip and knee. The same color coding applies to all figures. Please see Fig. 3 for more details.

However, on the other hand, there is a marked increase in the moments postintervention in the contralateral (left) limb. Figure 8, shows a view of the left knee and hip only. It shows an increase of the moments after the intervention in the left hip and knee. The changes are most obvious in the second half of the stance in the left knee in X (flexion/extension) and Y (valgus/varus) dimensions, and throughout the stance in the Z dimension (internal/external rotation). Although the kinematics view (Fig. 7) does not show comparable changes, OA can occur with unaltered kinematics^19^. A related study showed that mild kinematics changes can be detected in the early stage of knee OA such as a reduction in axial tibial rotation^20^. Besides, while a systematic review has shown that small sagittal plane kinematics emerge during stair climbing in OA^21^; non-sagittal kinematics do not appear to be neither markedly affected nor systematically affected in the same way across studies.

After observing the big picture, we can interpret the case as an example of early detection of predisposed osteoarthritis in the contralateral limb. The intervention improved (decreased) the patient’s moments on the right (affected) limb, but it led to adverse or inadequate weight bearing compensation on the left (contralateral) limb. Hence, this patient is at risk of developing osteoarthritis in his left limb in the future^22–24^, unless immediate preventive and/or corrective actions are taken.

The level of improvement, which we interpret to mean “effect magnitude” can be interpreted directly from the test statistic: the t-statistic for t-tests and the T^2^ statistic for Hotelling’s tests. However, if the effect as inferred from the t-statistic appears to be larger than the effect inferred from the T^2^ statistic then this is an illusion. The t-statistic considers variance along a single axis, and not the full 3D variance. The T^2^ statistic accounts for 3D variance, so if its effects appear to be smaller it is due to substantial 3D variance. This general perspective: that simple t-tests cannot capture complex multi-dimensional variance, is fundamental to multivariate statistics^25^ and is why we chose to present both multi- and univariate results.

### Benefits of MovementRx

To the best of our knowledge, no other software correctly model joints in 3 degrees of freedom with the multivariate approach. MovementRx employs SPM1D to analyze the extent of deviation a movement model (gait) has from a normal model. SPM1D can detect any curve differences (e.g., moments) with changing time, providing a qualitative and robust method of assessment of the locations of deviation and how much deviation from normal a given signal is. Therefore, using MovementRx solves the inconsistency problem in the gait analysis process and replaces it with a simple, coherent, objective, and intuitive GUI application.

MovementRx is opensource and it depends on open-source technologies. It overcomes the limitations of non-availability of CGA experts.

### Future plans

- “Region of Interest (ROI)”^26^ will be implemented to allow the statistics to be calculated only on part(s) of the stride, for the cases when the full data is not available or when the user is interested only in a part of the movement, e.g. the initial or terminal part.
- Registering the signal^27^ (warping to match reference peaks and valleys): Every person has his/her own gait style. Normalizing the signal in time may not be enough to align the peaks to the reference model, giving a false impression of deviation from normal. In this case, it would be beneficial to warp the signal to align its peaks to the reference. We will use mwarp1d library.
- Plugin tools will be added. The first plugin to consider will be an EMG analysis plugin.

## Conclusions

In this paper, we presented MovementRx, a simple, cascaded coherent, objective, and intuitive movement analysis GUI decision support system, using colormaps, especially directed for clinicians with limited statistical training, complimenting subjective assessments of whole-trajectory curves. No other software correctly model joints in 3 degrees of freedom using the multivariate analysis approach, nor present the deviation as colormaps in a cascaded view.

We presented a showcase of osteoarthritis with contralateral secondary osteoarthritis predisposition, that would pass undetected depending on the subjective assessment using the human eye.

## Supplementary Information

Supplementary Information.

## Data Availability

The reference normal data is from the ability data project^18^. The patient data is from a previous study in Australia. The data can be accessed within this project’s repository under the path “/res/cases”, or directly from the following link: https://github.com/aalhossary/MovementRx/tree/master/spmclient/res/cases. Any more information can be obtained from the corresponding author on reasonable request. Project name: MovementRx. Project home page: https://github.com/aalhossary/MovementRx. Operating system(s): Python (Platform independent) + different OS-specific executables. Programming language: Python 3.7 + Qt 5. Other requirements: *SPM1D, NumPy, SciPy, matplotlib, and PyQt5*. License: GNU General Public License v3 (GPLv3). Any restrictions to use by non-academics: GNU General Public License v3 (GPLv3).

## Abbreviations

CLI: Command line interface
GUI: Graphical user interface
SPM: Statistical parametric mapping
SPM1D: One-dimensional statistical parametric mapping
TKR: Total knee replacement
