# Supplementary material for: Versatile clinical movement analysis using statistical parametric mapping in MovementRx
Source: Sci Rep. 2023 Feb 10;13:2414. doi: 10.1038/s41598-023-29635-4 (PMC9918450; doi:10.1038/s41598-023-29635-4)
Supplement: Supplementary file 1 — Supplementary Information. [file 41598_2023_29635_MOESM1_ESM.docx]

Versatile clinical movement analysis using Statistical Parametric Mapping in MovementRx
(Supplementary Data)

# Authors

**Amr ALHOSSARY**^*1^, [aalhossary@ntu.edu.sg](mailto:aalhossary@ntu.edu.sg)

**Todsd Pataky**^2^, [pataky.todd.2m@kyoto-u.ac.jp](mailto:pataky.todd.2m@kyoto-u.ac.jp)

**Ang Wei Tech**^1^, [WTAng@ntu.edu.sg](mailto:WTAng@ntu.edu.sg)

**Karen Chua Sui Geok**^3^, [Karen_Chua@ttsh.com.sg](mailto:Karen_Chua@ttsh.com.sg)

**Wai Hang KWONG**^4^, [wai-hang.kwong@polyu.edu.hk](mailto:wai-hang.kwong@polyu.edu.hk)

**Cyril John Donnelly** †^1^, [cyril.donnelly@ntu.edu.sg](mailto:cyril.donnelly@ntu.edu.sg)

^1^ Rehabilitation Research Institute of Singapore (RRIS), Nanyang Technological University, Singapore

^2^ Kyoto University Graduate School of Medicine, Department of Human Health Sciences, Japan

^3^ Tan Tock Seng Hospital Rehabilitation Centre, Singapore

^4^ The Hong Kong Polytechnic University, Department of Rehabilitation Sciences

^*^ Corresponding author

† Passed away


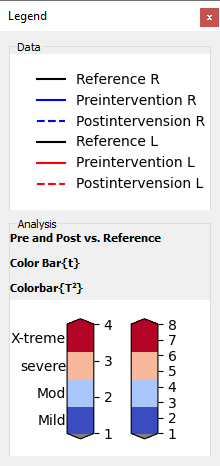


Supplementary Figure 1 Legend


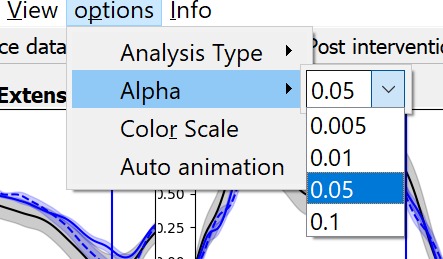


Supplementary Figure 2 Options menue with open Alpha menu item

Supplementary Table 1 Aggregate measurements of reference data

|  |  | **Velocity (m/s)** | **Height (m)** | **Weight (kg)** | **Age (yrs)** | **BMI (kg/m^2^)** | **Gender** |
| --- | --- | --- | --- | --- | --- | --- | --- |
| **Patient’s characteristics** | **Pre-TKR** | **1.22** | **1.73** | **77** | **55** | **25.7** | **M** |
|  | **Post- TKR** | **1.28** | **1.73** | **75** | **56** | **25.1** |  |
| **Characteristics of reference participant** | **Mean** | 1.38 | 1.68 | 69.7 | 61.3 | 24.5 | All are M |
|  | **Stdev** | 0.28 | 0.06 | 7.00 | 6.6 | 1.1 |  |
